# Peritoneal Dialysis in Acute Kidney Injury: Trends in the Outcome across Time Periods

**DOI:** 10.1371/journal.pone.0126436

**Published:** 2015-05-12

**Authors:** Daniela Ponce, Marina Berbel Buffarah, Cassiana Goes, André Balbi

**Affiliations:** University São Paulo State- UNESP, Distrito de Rubiao Junior, Botucatu, Sao Paulo, Brazil; University of São Paulo School of Medicine, BRAZIL

## Abstract

Peritoneal dialysis (PD) should be considered a suitable method of renal replacement therapy in acute kidney injury (AKI) patients. This study is the largest cohort providing patient characteristics, clinical practice, patterns and their relationship to outcomes in a developing country. Its objective was to describe the main determinants of patient and technique survival, including trends over time of PD treatment in AKI patients. This was a Brazilian prospective cohort study in which all adult AKI patients on PD were studied from January/2004 to January/2014. For comparison purposes, patients were divided into 2 groups according to the year of treatment: 2004-2008 and 2009-2014. Patient survival and technique failure (TF) were analyzed using the competing risk model of Fine and Gray. A total of 301 patients were included, 51 were transferred to hemodialysis (16.9%) during the study period. The main cause of TF was mechanical complication (47%) followed by peritonitis (41.2%). There was change in TF during the study period: compared to 2004-2008, patients treated at 2009-2014 had relative risk (RR) reduction of 0.86 (95% CI 0.77 – 0.96) and three independent risk factors were identified: period of treatment at 2009 and 2014, sepsis and age> 65 years. There were 180 deaths (59.8%) during the study. Death was the leading cause of dropout (77.9% of all cases) mainly by sepsis (58.3%), followed cardiovascular disease (36.1%). The overall patient survival was 41% at 30 days. Patient survival improved along study periods: compared to 2004-2008, patients treated at 2009-2014 had a RR reduction of 0.87 (95% CI 0.79 – 0.98). The independent risk factors for mortality were sepsis, age >70 years, ATN-ISS > 0.65 and positive fluid balance. As conclusion, we observed an improvement in patient survival and TF along the years even after correction for several confounders and using a competing risk approach.

## Background

In the 1970s, acute peritoneal dialysis (PD) was widely accepted for acute kidney injury (AKI) treatment, but its practice declined in favor of hemodialysis [1–5]. Recently, interest in using PD to manage AKI patients has been increasing. It is frequently used in developing countries because of its lower cost and minimal infrastructural requirements [4–5]. However, in developing countries the infrastructure for quality research is often lacking and the result has been limited evidence on standardized treatment regimes such as indications, dosing and volumes, and technical failure and mortality. The studies performed by Po7nce-Gabriel et al. [6] showed that, with careful thought and planning, critically ill patients can be successfully treated by PD. To overcome some of the classic limitations of PD use in AKI, such as a high chance of infectious and mechanical complications and no metabolic control, they proposed the use of cycles, flexible catheter, and a high volume of dialysis fluid. It is also true that PD is not the most efficient therapy: clearance per exchange can decrease if a shorter dwell time is applied, a lower efficiency can be observed in large-sized and severely hypercatabolic patients, fluid removal can be limited, and there is a high risk of infection [7–11]. Given the paucity of good-quality evidence in this important area, additional large cohorts studies on the use of PD for AKI and its effect on clinical outcomes are necessary. The present study is the largest representative cohort study providing this information in the world. Large cohorts provide the opportunity to assess a wide variety of both exposures and outcomes. The information acquired from these studies is particularly important in fields where randomized clinical trials are difficult to perform. Another potential advantage of the analysis of large longitudinal cohort studies is related to the possibility of providing an overview of trends in patient and treatment characteristics. Therefore, the objective of the present study was to describe the characteristics of the population, clinical practice patterns, and their relationship to clinical outcome in a large prospective cohort. In addition, we aimed to analyze temporal trends in technique survival and outcome of AKI patients treated with PD.

## Materials and Methods

### Study Population

This study was a prospective cohort study approved by the ethics committee of the Botucatu School of Medicine, Sao Paulo, Brazil and the participants (or their legal caregiver) provided their written informed consent to participate in it. Three hundred and one patients who had been consecutively treated by high volume PD were evaluated between January of 2004 and January of 2014. The inclusion criteria were AKI patients according to Acute Kidney Injury Network criteria [12], and severe acute tubular necrosis (ATN) caused by ischemic, nephrotoxic injury or clinical diagnosis of septic AKI [13].

Indications for dialysis were uremia or azotemia (BUN > 100 mg/dl), fluid overload (after diuretics use), electrolyte imbalance (K.6.5 mEq/L after clinical treatment), and acid–base disturbances (pH7.1 and bicarbonate10 mEq/L after clinical treatment). Exclusion criteria were under 18 years of age, functional azotemia (by clinical definition, the integrity of renal parenchyma tissue was maintained and glomerular filtration rate was corrected rapidly, in less than 24 h on restoration of renal perfusion and glomerular ultrafiltration pressure, with normal urine sediment and fractional excretion of Na <1%), urinary tract obstruction, acute interstitial nephritis, rapidly progressive glomerulonephritis (clinical criteria, defined as, absence of criteria for ATN, relevant clinical history and blood test with granular casts in urine sediment: red and white blood cells cast, proteinuria, or eosinophiluria >5%), a history of chronic kidney disease in advanced stages (IV and V), renal transplantation, pregnancy, severe hypercatabolism according to Schrier criteria [14], absolute contraindication for PD defined as recent abdominal surgery (less than one month), multiple abdominal surgeries (more than three), severe hyperkalemia with EKG changes, severe respiratory failure (FiO2> 70%) and severe fluid overload (pulmonary edema) [6,9,11]. If patients presented any one of these contraindications, they were treated by intermittent conventional, prolonged, or continuous hemodialysis (HD) according to their hemodynamic instability.

### Study Protocol

One high volume DP session was defined as 24 h with sessions performed 7 d/wk. Peritoneal access was established by the blind percutaneous placement of a flexible catheter using a Trocath-introduced paramedian from 2004 to 2008 and Seldinger technique from 2009 to 2014. Cephazolin was used as a prophylactic antibiotic to cover the PD catheter insertion. Patients were treated with continuous high volume PD, and exchanges with Dianeal PD solution (Na = 135 mEq/L,Ca = 3.5 mEq/L, K = 0 mEql/L, Mg = 1.5 mEq/L, lactate = 40mEq/L, 1.5%–4.25% glucose) were performed using HOMECHOICE cycler. The prescribed HVPD dose was determined by Kt/V [15], where K is the volume of dialysis solution prescribed in 24 h (in milliliters) x 0.6 (considering the relationship of urea nitrogen (UN) dialysate/plasma = 0.6 in 1 hour), t is treatment duration (1 day), and V is the volume (in liters) of body urea distribution by the formula by Watson et al [16]. The prescribed Kt/V value ranged from 0.6 to 0.8/session (mean was 0.67±0.11) from 2004 to 2008 and 0.5 to 0.65(mean was 0.57±0.06) from 2009 until 2014; 1.5 to 2-L exchanges were performed with 30–60 minutes of dwell time (total of 32–44 L/d and 16–30 exchanges/d). To evaluate the adequacy of the dialysis, daily delivered Kt/V, ultrafiltration (UF), and patients’ catabolic rates were calculated. The delivered high volume PD dose was determined by urea Kt/V, where K was the mean dialysate UN in milligrams per 100 milliliters/plasma UN pre- and post dialysis in milligrams per 100 milliliters X drained volume in 24 h in milliliters/volume of body urea distribution in milliliters [15,17]. Blood samples were collected at the beginning and end of each HVPD dialysis session and analyzed for creatinine, potassium, bicarbonate, glucose, and sodium levels. One aliquot of 3 ml of all the spent dialysate was collected in every session to measure UN. Dialysate white blood cell count and cultures were also determined every 3 days.

Patients’ catabolic rate was calculated based on nitrogen balance (NB), and patients were classified according to their NB as being mildly hypercatabolic (NB< 6 g/d), moderately hypercatabolic (NB between 6 and 12 g/d), and severely hypercatabolic patients (NB>12 g/d) [18]. Dietary protein was calculated from a 24-h dietary intake of patients closely supervised by a renal dietician. Eight patients were excluded from the NB calculation, because they received an oral diet, leading to an unreliable measurement of protein intake. Anthropometric measurements (weight, height, and body surface area) were obtained before dialysis. Body surface area was calculated from the Du Bois formula [19]. Mobile patients were weighed on a digital scale, and the weight of immobilized patients was obtained by a bed scale or calculated from two variables formula [20]. Other variables, including the etiology of AKI, urine output, number of dialysis sessions, need for mechanical ventilation, presence of hemodynamic instability, and patient outcome, prognostic index of AKI evaluated by Acute Tubular Necrosis Individual Severity Score (ATN-ISS) [21], were analyzed. The ATN-ISS is a linear discriminant model producing a percent likelihood of mortality on the basis of a demographic data, causes of ATN, urine output, need for dialysis and clinical conditions.

The protocol was interrupted when there was partial recovery in renal function (urine output>1000 ml/d) and progressive drop in creatinine (<4 mg/dl) and BUN levels (<50 mg/dl), need to change dialysis method because of infectious and mechanical complications, or inefficacy of HVPD in failing to remove fluid and solute, treatment for more than 30 days of follow-up or death.

For the analysis of technique survival, the primary event was defined as transfer to hemodialysis (HD) for any reason, which means the patient did not return to PD until the end of the follow-up. Dropout data were stratified as death, recovery of renal function, and transfer to HD. For the description of trends in population characteristics, patient and technique survival, the population was divided into two groups according to the year of PD treatment: 2004 to 2008 and 2008 to 2014.

### Statistical Analyses

Results are presented as mean and standard deviation (SD) or median and range according to normality characteristics for each variable with a 5% significance level; t test was used to compare parametric variables between two groups and ANOVA followed by the Newman–Keuls test for multiple comparisons between groups. For nonparametric variables, Wilcoxon and Kruskal–Wallis tests followed by Dunn’s method were used to compare two groups and multiple groups, respectively. For analysis of repeated measures, the Proc mixed program was used.

Categorical variables were expressed as proportions and compared with the chi-squared test. Variables with p less than 0.20 in univariate associations were candidates for multivariable analysis.

We use a competing risk model based on Fine and Gray [22] for adjusted multivariate patient and technique survival. A competing risk is an alternative outcome that is of equal or more significant clinical importance than the primary outcome and alters the probability of the outcome of interest. That is, we limit our analysis to the consideration of two (competing) events: technique failure and deaths; deaths occurring after the TF are not considered in this analysis.

As opposed to a cause-specific analysis, which would censor the competing event(s), the Fine-Gray approach “carries forward” the competing event(s) in the risk set, with appropriate weighting, and does not censor them. The Fine-Gray model assumes proportional hazards, and because of inferential problems associated with the incorporation of time-varying covariates when death is a competing event, such covariate function was utilized in a heuristic sense to demonstrate nonproportional hazards [22].

All patients active at the end of follow-up were treated as censored.

All analysis was adjusted for covariates. Finally, collinearity among variables was tested and if statistically significant interactions were present, 1 of them was excluded. Covariates were included in the model when a *p* value lower than 0.20 in the univariate analysis was found. Statistical significance was set at *p* < 0.05. All statistical descriptive analyses were performed with SPSS 20.0 (SPSS, Chicago, USA). The competing risk analysis was performed using STATA 12 (StataCorp LP, College Station, TX, USA) and the package cmprsk: Subdistribution Analysis for Competing Risks, R version3.0.2 (R Foundation for Statistical Computing).

## Results

During the study period (10 years), a total of 1231 patients were treated by dialysis: 301 by high volume PD (24.4%) and 930 by HD (75.6%), of which 323 were treated by conventional, 519 were treated by prolonged, and 88 continuous HD.

In Table 1, we present the main characteristics of the general study population. The mean age was 66.8 ±15.7 years, 210 (69.8%) patients were male, 245 (81.4%) of patients were Caucasian, and the mean patient weight was 71.6 ±10.7 kg (148 (49.2%) were obtained by a digital scale, 101 (34.9%) were obtained by bed scale and 52 (17.3%) were calculated from two variable formulas).

**Table 1:** Clinical data of acute kidney injury patient treated with high volume peritoneal dialysis according to time period of high volume peritoneal dialysis treatment.

	General	2004–2008	2009–2014	P
	(n = 301)	(n = 168)	(n = 133)	
**Age (years)**	66.8±15.7	64.7±15.1	69.8±15.9	0.04
**Male sex (%)**	210 (69.8)	120 (71.4)	91 (68.4)	0.33
**Caucasian patients (%)**	245 (81.4%)	33 (79.2)	111(83.4)	0.51
**Weight (kg)**	71.6±10.7	70.1±10.1	72.5±10.9	0.43
**ICU (%)**	201 (66.8)	119 (70.8)	80 (60.2)	0.05
**Dialysis indication**				
Azotemia (%)	190 (63.1)	110 (65.5)	83 (62.4)	0.81
Hyperkalemia (%)	39 (12.6)	22 (13.1)	14 (10.5)	0.53
Fluid overload (%)	61 (20.3)	34(20.2)	27(20.3)	0.59
Others * (%)	11(3.6)	6 (3.5)	5(3.8)	0.85
**Main diagnosis**				
Sepsis (%)	166(55.1)	105 (62.5)	62 (46.6)	0.04
Cardiovascular (%)	78 (25.9)	39 (23.2)	41 (30.8)	0.11
Others** (%)	57 (18.9)	24(14.3)	30 (22.6)	0.77
**AKI etiology**				
Sepsis (%)	160 (53.2)	104 (61.9)	63 (47.4)	0.04
i ATN (%)	81 (26.9)	40 (23.9)	42 (31.6)	0.09
Others *** (%)	60(19.9)	24 (14.3)	25 (18.8)	0.31
**ICU admission (%)**	201 (66.8%)	124(73.8)	84 (63.1)	0.08
**Mechanical ventilation (%)**	213 (70.3)	121 (72)	85 (63.9)	0.23
**Vasoactive drugs (%)**	189(62.8)	108 (64.3)	81(60.9)	0.31
**Urine output (<400 ml/day)**	205 (68.1)	111 (66.1)	91 (68.4)	0.44
**ATN-ISS**	0.63±0.18	0.66±0.19	0.62±0.16	0.08
**Number of sessions (days)**	7 (5–11)	6 (5–10)	7 (6–12)	0.19
**Follow-up (days)**	9 (6–14)	8 (5–13)	10 (6–14)	0.11
**Mortality after 30 days (%)**	180 (59.8)	111 (66.1)	69 (51.9)	0.049

ICU: intensive care unit, i ATN: ischemic acute tubular necrosis, ATN-ISS: acute tubular necrosis individual severity score

* others: acidosis, more than one indication;

** others: liver diseases and post surgery;

*** others: nephrotoxic AKI, obstructive AKI and mixed AKI

Most of the patients (66.8%) were in the intensive care unit and needed vasoactive drugs and mechanical ventilation (62.8% and 70.3%, respectively); 205 patients (68.1%) had low urine output (less than 400ml/day), and the mean ATN-index specific score (ISS) was 0.63 ±0.18. Sepsis was the main cause of AKI (53.2%) followed by heart failure (26.9%). Uremia or azotemia was the main indication for dialysis (63.1%). The median number of high volume sessions was 7, with a range of 5–11 and the delivered urea Kt/V was 0.56±0.18/session and 3.9 ±0.8/wk.

Table 1 also shows the characteristics of the study population divided into two different time periods of PD treatment. Over the years, the prevalence of septic AKI patients treated with PD decreased from 61.9 to 47.4% (p = 0.04) and there was a reduction in the prevalence of patients admitted to intensive care unit (ICU) from 73.8 to 63.1% (p = 0.05). It also occurred an increase in age of patients treated by PD from 64.7±15.1 to 69.8±15.9 years old (p = 0.04).

Table 2 shows the general metabolic control and fluid balance after high volume PD initiation. BUN and creatinine levels stabilized after four sessions, and bicarbonate and pH levels stabilized after three sessions. The mean UF increased steadily from one to three sessions and stabilized after four sessions at around 1.300 ml/d.

**Table 2:** Prescription and metabolic and fluid control of acute kidney injury patient treated with high volume peritoneal dialysis according to time period.

	Overall(n = 301)	2004–2008(n = 168)	2009–2014(n = 133)	P
**Pre BUN (mg/dl)**	113.7±28.3	118.7±29.3	111.7 ± 21.9	0.56
**Pre creatinine**	5.7 ± 2.8	5.9 ± 2.9	5.4 ± 2.3	0.78
**BUN after (mg/dl)**				
1st session	95±42	95±42	89±32	0.34
2nd session	86±31	86±31	79±21	0.29
3rd session	71±27	73±28	69±19	0.39
4th session	62±18	64±18	56±22	0.41
5th session	54±15	55±15	51±11	0.53
**Creatinine after (mg/dl)**				
1st session	5.3±1.5	5.1±1.5	5.4±1.6	0.64
2nd session	4.7±1.2	4.6±1.2	4.9±1.4	0.61
3rd session	4.5±1.3	4.3±1.3	4.7±1.5	0.76
4th session	4.0±1.1	3.9±1.1	4.2±1.3	0.47
5th session	3.8±1.1	3.7±1.1	4.1±1.2	0.71
**Bicarbonate (mEq/l)**				
1st session	17.4±4.7	16.4±4.7	18.1±4.9	0.54
2nd session	20.8±4.5	20.1±4.3	21.1±4.7	0.59
3rd session	21.6±3.5	21.2±3.5	21.9±4.5	0.69
4th session	22.6±3.5	22.5±3.4	22.8±4.4	0.71
5th session	22.2±3.6	22.8±3.2	23.7±4.1	0.77
**UF (ml/session)**				
1st session	275 (-370–515)	230 (-330–465)	344 (-87–565)	0.14
2nd session	989.8 (693.4–1221.8)	919.8 (683.4–1119.8)	1015 (756–1348)	0.09
3rd session	1183 (901.4–1523)	1023 (800.4–1323)	1405 (1018–1768)	0.04
4th session	1512.9 (1070–1602)	1102.9 (870–1402)	1976 (1422–2099)	0.01
5th session	1700.6 (1158–1970)	1200.6 (958–1570)	2012 (1865–2314)	0.018
**FB after (g/day)**				
1st session	0.2 ±0.04	0.5±0.1	-0.1 ±0.02	0.37
2nd session	-1.4±0.1	-0.8±0.3	-1.7 ±0.5	0.19
3rd session	-1.9±0.4	-1.1±0.4	-2.2±0.6	0.03
4th session	-2.1± 1.9	-1.4 ±0.5	-2.9± 0.9	0.008
5th session	-2.2 ± 1.1	-1.2 ±0.4	-2.3± 0.6	0.001
**Prescribed Kt/V**Per session				
Per session	0.62±0.01	0.67±0.11	0.57±0.06	0.03
Weekly	4.34±0.01	4.7±0.71	3.9±0.51	0.04
**Delivered Kt/ V**				
Per session	0.56±0.15	0.58±0.18	0.49±0.14	0.04
Weekly	3.9 ± 0.8	4.0 ± 0.9	3.4 ± 0.7	0.02

Metabolic control and fluid balance of the study population divided into two different time periods of PD treatment are also shown in Table 2. The prescribed and delivered dialysis PD dose by decreased 17.9 and 15.5%, respectively, while there was no difference in metabolic control. There was an increase in cumulative negative fluid balance (defined as superior to 0 ml after 2 session of PD) and UF over the years after 3 PD sessions.

### Technique survival

Peritonitis occurred in 31 patients (10.3%) after 5.8±1.6 high volume PD sessions. Twenty-one patients (67.8%) had the catheter removed, and the dialysis method was changed because of no improvement in laboratory or clinical parameters after 5 days of correct antibiotic treatment. The main agents were Pseudomonas aeruginosa, (14 cases, 45.1%), fungi (7 cases, 22.6%), Staphylococcus aureus (5 cases, 16.1%) and acinetobacter (3 cases, 9.7%). Antibiotic treatment was maintained from 14 to 21 days. Forty four patients presented mechanical complications (14.6%) and leakage and tip catheter migration were the majority of the complications (79.5%), with therapy being interrupted in 24 patients (54.5%). In other cases, the catheter was reinserted, and the dialysate volume per cycle was reduced (1200–1500 ml/cycle).

**Fig 1:**
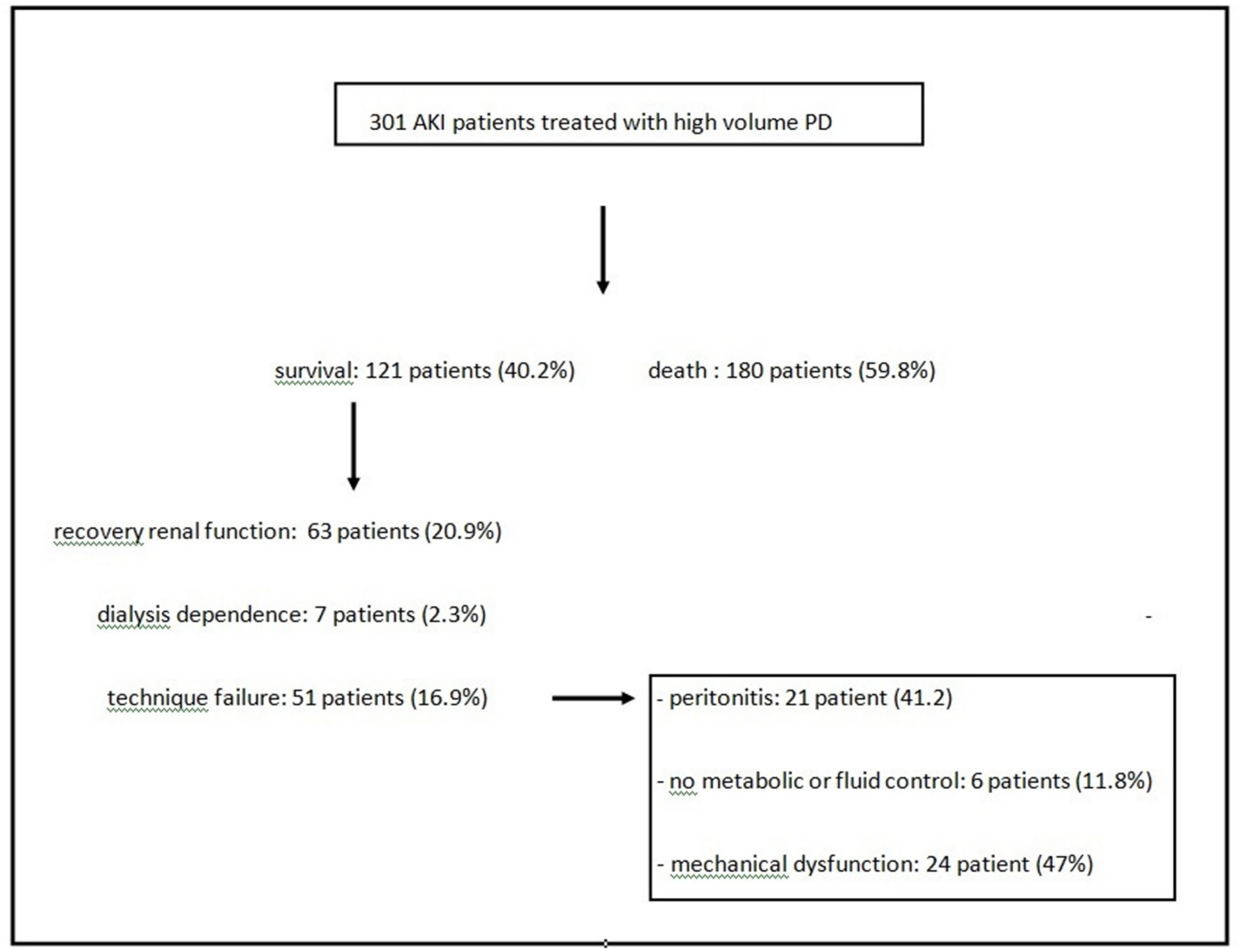
Acute kidney injury (AKI) patient outcome treated with high volume peritoneal dialysis.

Out of 301 patients, 51 were transferred to HD (16.9%) during the study period. The main cause of technique failure was catheter dysfunction (47%) followed by peritonitis (41.2%), low ultrafiltration rate (5.9%) and no metabolic control (3.9%). The technique survival after 30 days was 84%. Death was responsible for more than 50% of the study dropout with mechanical complications coming next. Table 3 summarizes the infectious and mechanical complications related to PD and Table 4 shows the causes of dropout. Mechanical complications were the most important cause of technique failure followed by peritonitis as shown in Fig 1.

**Table 3:** Mechanical and infectious complications related to high volume peritonealdialysis according to time period.

PD complications	Overall(n = 301)	2004–2008 (n = 168)	2009–2014 (n = 133)	p
Peritonitis (%)	**31 (10.3)**	**21 (12.5)**	**10 (7.5)**	**0.09**
Mechanical (leakage and catheter tip migration (%)	**44 (14.6)**	**31 (18.5)**	**13 (9.8)**	**0.04**

**Table 4:** Dropout causes according to time period.

Dropout causes		Death censured		
	overall	2004–2008	2009–2014	p
	(n = 51)	(n = 37)	(n = 14)	
**catheter dysfunction (%)**	25 (49)	19 (51.4)	6 (42.8)	0.049
**Peritonitis (%)**	21 (41.2)	17 (46)	4 (28.6)	0.037
**No metabolic or fluid control (%)**	5 (9.8)	1 (2.7)	4 (28.6)	0.02

**Fig 2:**
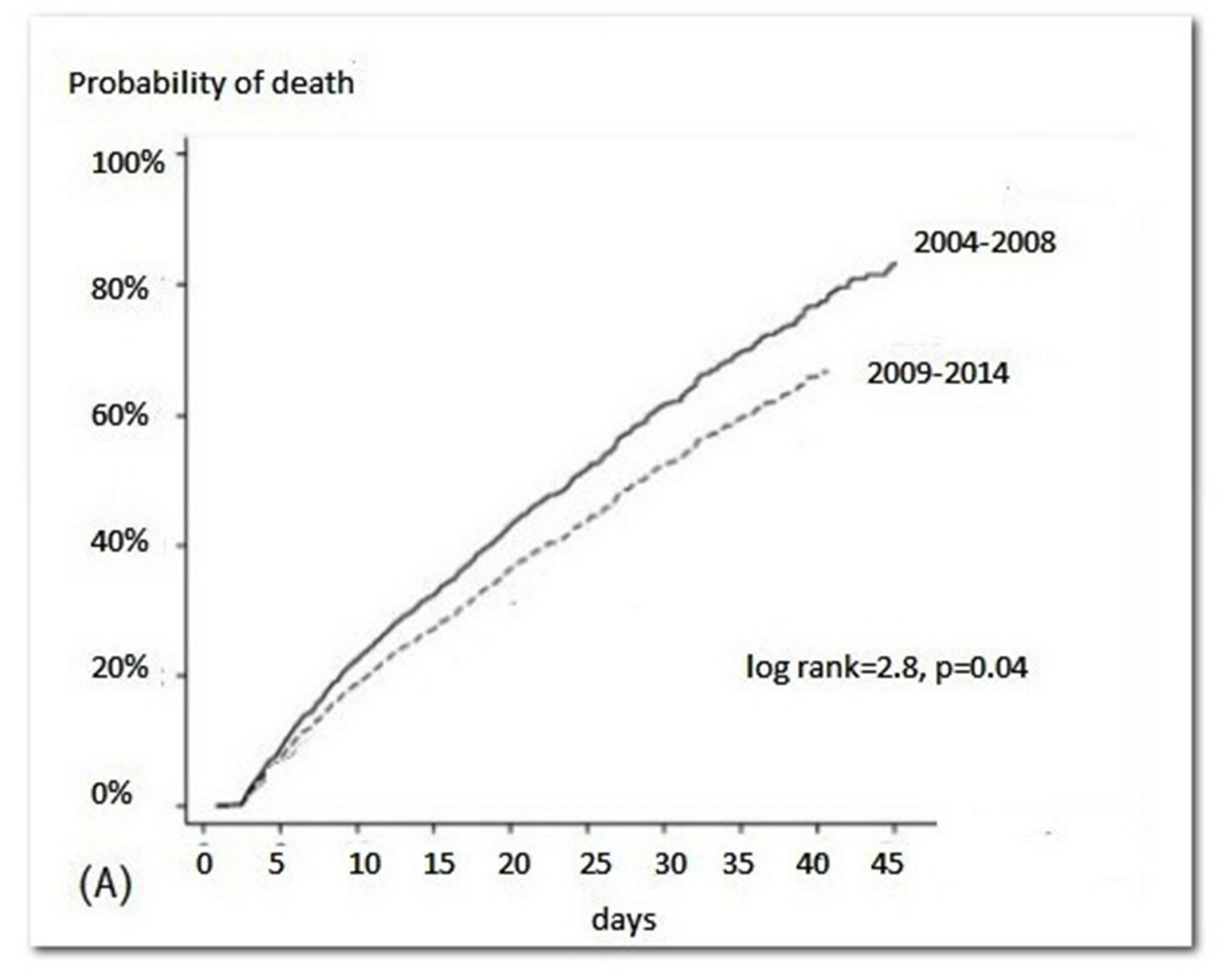
Probability of death according to time period.

There was change in TF during the study period: compared to 2004–2008, patients treated at 2009/2014 had a relative risk reduction of 0.86 (95% confidence interval [CI] 0.77–0.96) (Fig 2). Five covariates presented a *p* value lower than 0.20 in the univariate analysis and were included in the multivariate model: age ≥ 65 years, sepsis, ATN-ISS > 0.65, ICU admission and period of treatment. Three independent risk factors were identified: sepsis, period of treatment from 2004 to 2008 and age > 65 years (Table 5).

**Table 5:** Subdistribuition Hazard Ratio of Covariates for technique failure.

Variables	HR (CI95%)	p
**2004–2008**	ref	
**2009–2014**	0.92 (0.87–0.97)	0.01
**Age > 65 years**	1.26 (1.04–1.39)	0.049
**ATN-ISS > 0.65**	1.11 (0.98–2.11)	0.29
**Sepsis**	1.32 (1.18–2.64)	0.02
**ICU admission**	1.12 (0.98–1.63)	0.12

### Patient survival

Concerning patient outcome, (63) 20.9% of patients recovered renal function, whereas (7) 2.3% were kept on dialysis after 30 days of therapy. Change of the dialysis method occurred in 16.9% of patients because of refractory peritonitis or mechanical complications (leakage or UF failure).

There were 180 deaths (59.8%) during the study. In fact, death was the leading cause of dropout (77.9% of all cases) mainly by sepsis (58.3%), followed cardiovascular disease (36.1%).

**Fig 3:**
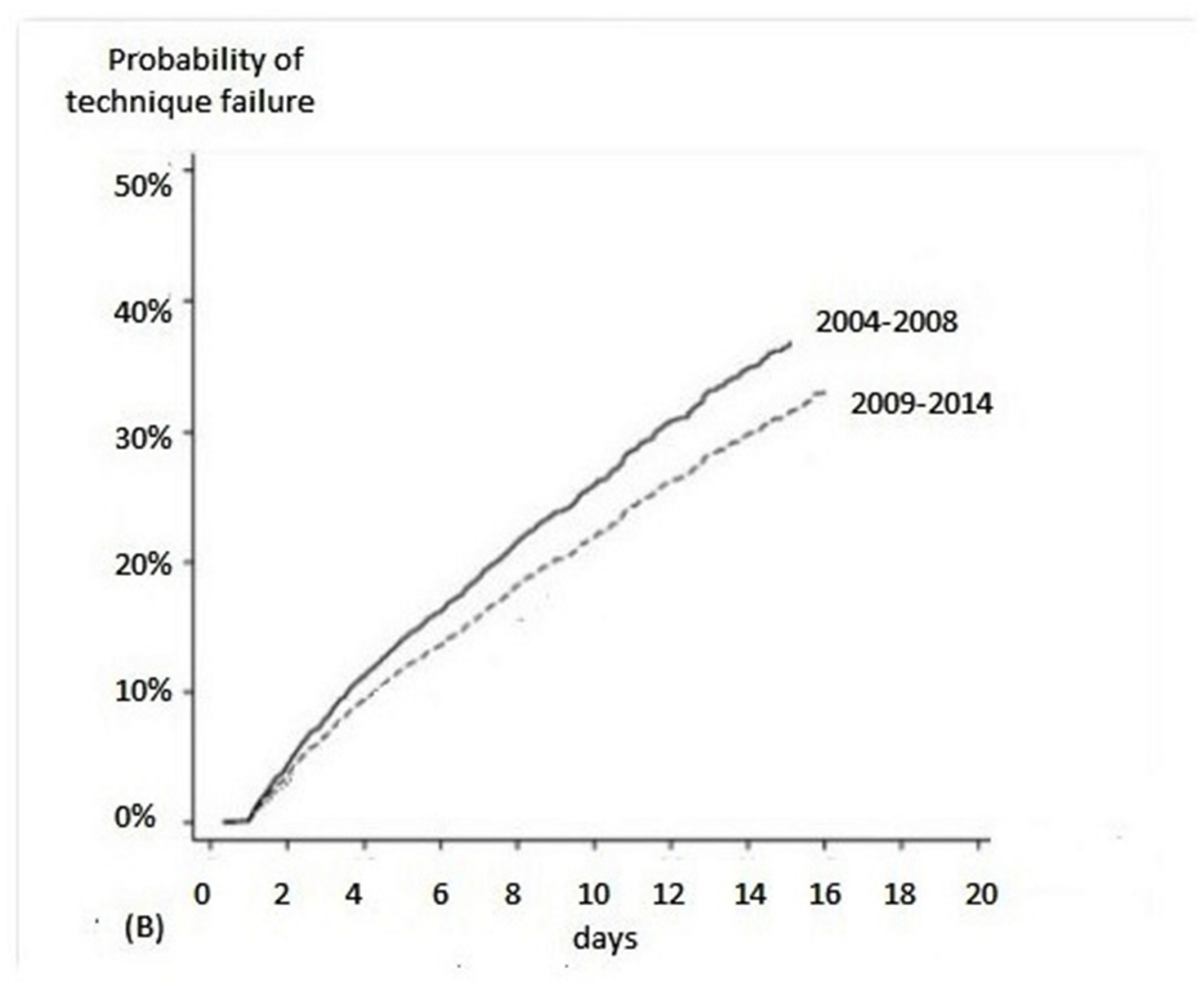
Probability of technique failure according to time period.

Overall no adjusted patient survival at 41% after 30 days. Mortality rates improved over the years: compared to 2004 to 2008 patients, those treated at 2009 to 2014 had a relative risk reduction (HR) of 0.87 (95% CI 0.79–0.98) (Fig 3). After the inclusion of all variables with a *p* value lower than 0.20 at the univariate analysis (age ≥ 70 years, sepsis, ATN-ISS > 0,65, low urine output, ICU admission, UF < 500 ml/day, positive fluid balance and the year the patient treated by PD) we ended up with 6 independent predictors of mortality: age ≥ 70 (HR: 2.44, 95% CI 2.15–2.77), ICU admission (HR 2.12, 95% CI 1.67–3.64), ATN-ISS > 0.65 (HR: 1.18, 95% CI, 1.03–1.35), sepsis (HR: 1.26, 95% CI, 1.10–1.45), positive fluid balance (HR:1.58,95% CI, 1.46–2.73) and the year the patient treated with PD (2004 to 2008 compared to 2009 to 2014: HR: 0.87, 95%CI, 0.79–0.98). A full description of patient survival according different subsets of patients can be seen in Table 6.

**Table 6:** Subdistribuition Hazard Ratio of Covariates for mortality.

Variables	HR (CI95%)	p
**2004–2008**	ref	
**2009–2014**	0.94 (0.89–0.98)	0.01
**Age > 70 years**	2.44 (2.15–2.77)	0.009
**ATN-ISS > 0.65**	1.18 (1.03–1.35)	0.04
**Sepsis**	1.26 (1.10–1.45)	0.02
**ICU admission**	2.12 (1.67–3.64)	0.003
**Low urine output**	1.17 (0.94–1.49)	0.09
**UF < 500 ml/day**	1.19 (0.91–1.54)	0.25
**positive fluid balance**	1.58 (1.46–2.73)	0.008

## Discussion

Observational studies provide valuable information in patient selection, clinical practice, and their relationship to patient and technique outcome. The present study is the largest cohort about PD in AKI that showed the improvement in AKI patient survival treated with PD and in its TF.

The demographic characteristics of our PD patients changed over time, we noted a reduction in septic patients, an increase in age and a decrease in patients admitted to ICU.

These changes in our patient profile likely reflect a change in clinical practice probably based on emerging data pointing to worse outcomes of septic and severe hypercatabolic patients treated with PD [23,24]. According to Chitalia [23], Phu [25] and Ponce et al [24], hemodialysis should be the first option of treatment for severe hypercatabolic septic patients because PD clearance is limited by dialysate flow, membrane permeability, and area and cannot be enough to keep adequate metabolic and fluid control of these patients.

However, comparing our population with other but small studies cohorts, we still have a higher percentage of elderly, septic and critically AKI patients treated with PD when compared to the African and Indian cohorts. The mean age and the number of septic patients of our cohort were in average 15 years older and 30% more frequent than most others PD cohorts, respectively [23,26,27].

In terms of PD doses, there was a decrease in prescribed and delivered Kt/V, without changes in metabolic control. There was significant reduction in BUN and creatinine levels, with stabilization of BUN values (around 55 mg/dl) and bicarbonate (around 23 mEq/l) after four sessions in both periods of study. The most appropriate dose for PD in the management of patients with AKI is poorly defined. This reduction in prescribed doses reflects the results of previous studies [28,29]. Ponce et al have compared very high volume (prescribed Kt/V = 0.8) with lower volume acute PD (prescribed Kt/V = 0.5) and have shown no benefit from aiming for the higher target; the lower-dose group achieved a Kt/V urea of 3.43 and did as well as the higher-dose group, which achieved a Kt/V of 4.13 [29]. However, some studies have shown very good outcomes with much lower doses than those used in Ponce-Gabriel’s study [23,27] and the ideal delivered PD doses can be lower than that reported by Ponce et al [28,29]. There was an increase in cumulative negative fluid balance and UF over the years after 3 PD sessions. These results reflect results of previous studies reporting that low urine output and fluid overload were associated with worse prognosis of AKI patients [6,30,31].

### Technique survival: main findings

Traditionally, peritonitis is the leading cause of TF in chronic PD patients, which was different in the present study. In fact, mechanical complications (tip catheter migration) were the first cause of TF, followed by peritonitis. In the most cases of leakage, dialysate volume per cycle was reduced from 2000 ml to 1200 or 1500 ml/cycle and PD could be continued.

Concerning infectious complications, peritonitis levels were similar to those reported in the literature (12%–15%) and fungi and Pseudomonas aeruginosa were the most common agents [3,6,8,17]. Previous studies reported lower prevalence of mechanical complications; however they excluded patients who had them in the first 24 hours of PD treatment [3,6].

There was change in TF during the study period: compared to 2004–2008, patients treated at 2009/2014 had a relative risk reduction of 0.86 (95% confidence interval [CI] 0.77–0.96) and three independent risk factors were identified: period of treatment at 2009 and 2014, sepsis and age > 65 years. We also can observe that there was change in causes of TF over the years: a decrease in peritonitis and catheter dysfunction and an increase in no metabolic and fluid control. There is no data in the literature to be comparable to our study. We believe that the impact of period of study occurred because the team achieved more experience in catheter insertion and cycler preparation. Exploring how the sepsis can be risk factor to TF, it is known that these patients are, frequently, severe hypercatabolic that may impair metabolic and fluid control by PD, leading to dropout study [3,23,31].

### Mortality

Despite all efforts, mortality rates remain extremely high in dialysis AKI patients [32–37]. Not surprisingly, death was responsible for more than 50% of the study dropout and a survival patients after 30 days was 40%. In line with previous reports, the mortality of AKI patients undergoing different methods of dialysis ranged from 40–80%, according to AKI etiology and severity of patients [33,37].

In this study, mortality rates improved over the years: compared to 2004 to 2008 patients, those treated at 2009 to 2014 had a relative risk reduction (HR) of 0.87 (95% CI 0.79–0.98) and more five independent predictors of mortality were identified: age ≥ 70 years, ICU admission, ATN-ISS > 0.65, sepsis and positive fluid balance. These results agree with previous studies reporting that higher age, fluid overload, and sepsis were associated with worse prognosis of AKI patients [32–34,38].

Recent studies have shown that fluid overload is a risk factor of death in critical patients [30,31,38]. In chronic dialysis patients treated with automated PD, the work by Brown et al. [31] showed that UF lower than 750 ml/d was associated with death. Ponce et al [6], in study that evaluated 150 AKI patients treated with high volume PD, showed that higher age, sepsis, lower urine output, ultrafiltration lower than 500 ml/day after three sessions were identified as risk factors for death.

Finally, the improvement occurred in patients treated at 2009 to 2014 presenting better survival than those from 2004 to 2009. At first glance the better clinical profile of patients starting dialysis could be an explanation, but the difference persisted even after adjustment for several covariates. The factors responsible for this improvement were not clear, but are probably related to an improvement in clinical practice including better fluid control. Another possibility is related to a better management of PD-related infections that has been massively tackled by medical societies through development of campaigns and development and diffusion of clinical guidelines. Importantly, our results are in line with previous data in AKI patients from large cohorts that looked into secular trends: Waikar et al. in a large study with AKI patients in dialysis reported a significant improvement ranging from 3 to 5% in 2 to 5 years patient survival [37].

This study presents several limitations. First, this is an observational study and, as such, all significant associations found should be interpreted with caution. Second, residual renal function was not available for the majority of patients and was not included in our analysis. Nevertheless, our study has some very important strengths: it was a prospective, cohort with outcomes adjusted for several clinical covariates using a competing risks analysis. Its characteristics share several similarities with other cohorts from different parts of the world supporting the quality of our data.

In conclusion, we described the largest cohort of PD in AKI patients in the world. This is the first study reporting a significant trend in TF and patient survival improvement throughout the vintages in a developing country and after adjusting for multiple covariates.
